# Identification of lncRNA *HCCAT5* as a Novel Biomarker for Gastric Cancer

**DOI:** 10.1155/ijog/9917434

**Published:** 2025-08-13

**Authors:** Nazila Saedi Alvaroliya, Seyedeh Hayla Jahanshahi, Nazli Khajehnasiri, Fatemeh Zeinali Sehrig, Adel Abdi

**Affiliations:** ^1^Department of Biological Sciences, Faculty of Basic Sciences, Higher Education Institute of Rab-Rashid, Tabriz, Iran; ^2^Department of Biology, Islamic Azad University, Tabriz Branch, Tabriz, Iran; ^3^Department of Animal Biology, Faculty of Natural Sciences, University of Tabriz, Tabriz, Iran

**Keywords:** biomarker, gastric cancer, *HCCAT5*, lncRNA

## Abstract

**Background:** Gastric cancer (GC) is a widespread type of cancer on a global scale, standing as the fifth most frequently occurring malignant disease. Dysregulation of long noncoding RNAs (lncRNAs) due to genomic mutations in noncoding DNA sequences can contribute to the development of tumors. Detecting GC at an early stage can significantly improve the chances of survival and treatment effectiveness. The current study evaluated the expression of lncRNA *HCCAT5* in primary gastric tumors as well as in adjacent noncancerous tissues.

**Methods:** One hundred pairs of GC and adjacent noncancerous tissue samples were acquired from Tabriz International Valiasr Hospital in Iran for the study. Subsequently, RNA extraction was conducted, and this was followed by cDNA synthesis. The expression of *HCCAT5* was evaluated using quantitative reverse transcriptase PCR (qRT-PCR). The relationship between clinicopathological characteristics and *HCCAT5* expression was analyzed using SPSS software. Furthermore, the predictive value of *HCCAT5* in GC was examined via analysis of the receiver operating characteristic (ROC) curve.

**Results:** *HCCAT5* exhibited a significant upregulation in tumor samples in contrast to adjacent noncancerous tissues (*p* < 0.0001). On the other hand, no significant relationship was detected between the elevated expression of *HCCAT5* and the clinicopathological features of the individuals (*p* > 0.05).

**Conclusions:** The lncRNA *HCCAT5* is implicated in advancing the development of GC. Consequently, it could be considered a potential target for therapeutic interventions and a prognostic biomarker for GC patients.

## 1. Introduction

Gastric cancer (GC) is a highly lethal illness with unfavorable survival rates globally. The Asian and South American countries witness a significant number of new cases of GC each year, making them the primary regions for the majority of diagnoses [1]. Over 70% of these instances transpire in developing nations, particularly in East Asia [2]. GC is a prevalent form of cancer that is frequently diagnosed on a global scale [3–5]. This disease has resulted in significant challenges and burdens [3–5]. Despite the utilization of radiotherapy and perioperative chemotherapy, the prognosis of patients with GC continues to be unfavorable [6, 7]. A thorough comprehension of the molecular pathway of GC aids in the advancement of successful treatment strategies and enhances the prognosis of patients with GC [8]. Environmental and genetic factors are thought to influence the development of GC. Alterations in specific tumor suppressor and oncogene genes ultimately result in disrupted cellular proliferation, migration, and invasion [9, 10]. Despite notable progress in the detection and management of GC, the outlook for individuals affected by this condition remains predominantly bleak [11]. Hence, it is imperative to uncover the intricate molecular mechanisms underlying the oncogenesis and progression of GC in order to devise more efficacious prognostic strategies [12].

Long noncoding RNAs, or lncRNAs, are RNA molecules exceeding 200 nucleotides in length [13]. Distinguished by their molecular structure, these RNAs lack the open reading frame (ORF), thereby preventing them from encoding proteins [13, 14]. There is mounting evidence indicating that lncRNAs have significant involvement in a wide range of biological processes, such as gene regulation, protein stabilization, and mRNA splicing [15, 16]. Furthermore, lncRNA has the potential to function as both tumor suppressor genes and oncogenes, exerting influence on various cellular processes such as apoptosis [17], cell proliferation, metastasis, differentiation, DNA damage, immune response, angiogenesis, and other related mechanisms [18]. lncRNAs constitute a significant portion of the entire genome and exhibit distinct expression patterns in GC [19]. lncRNAs play a crucial role in governing the biological activities of GC cells, including proliferation, metastasis, and invasion, by acting as oncogenes or tumor suppressor genes. These lncRNAs exert their influence through diverse signaling pathways [20–22]. Numerous pieces of evidence have indicated that lncRNAs are intricately linked to the development and advancement of malignancies as well as the resistance of tumors to drugs. These lncRNAs play a significant role as indicators of tumor characteristics [23–25]. The lncRNAs have undergone thorough investigation as potential biomarkers for the diagnosis and prognosis of various cancers, such as liver cancer, lung cancer, renal cell carcinoma, cervical cancer, breast cancer, stromal sarcoma, uterine endometrial cancer, and colorectal carcinoma [26]. Nevertheless, the precise lncRNAs linked to GC are still not fully comprehended [26].

Hepatocellular carcinoma-associated transcript 5 (*HCCAT5*), also known as *HTA*, is a newly discovered gene linked to tumors. It was identified through cDNA xProfile, a computational tool that analyzes the EST database from the Cancer Genome Anatomy Project (CGAP) website. RT-PCR demonstrated that *HCCAT5* exhibited specific expression in particular tumor types, with a notably high expression level observed in hepatocellular carcinoma [27]. The *HCCAT5* gene is an innovative tumor-associated gene. To date, there has been a lack of extensive research conducted on the *HCCAT5* gene [27]. The knockdown experiment provided insights into the specific expression characteristics and cancer-promoting effect of *HCCAT5*, indicating its potential involvement in the development of hepatocellular carcinoma [28]. In this current investigation, we examined the levels of *HCCAT5* expression in GC and explored its potential correlation with the clinicopathological characteristics of the patients involved.

## 2. Materials and Methods

### 2.1. Samples

A total of 100 pairs of gastric tumors and nontumor tissues were gathered from Tabriz International Valiasr Hospital in Iran. The study was granted approval by the Medical Ethics Committee at the University of Tabriz and was conducted in accordance with the Declaration of Helsinki. All patients have given their written consent after being fully informed. Tissue samples were carefully collected in microtubes that were free from RNase and DNase, rapidly frozen in liquid nitrogen, and subsequently stored at a temperature of −80°C until they were required for further analysis.

### 2.2. RNA Extraction and cDNA Synthesis

TRIZOL reagent (Invitrogen, Massachusetts, United States) was utilized to extract total RNA from both GC tissues and adjacent normal specimens. RNA integrity was verified using agarose gel electrophoresis as well as spectrophotometric analysis (A260/A280 ratio) to ensure that all samples met accepted standards of purity and quality. DNase I (Thermo Fisher Scientific, Waltham, United States) was utilized on the RNA samples in order to remove any potential DNA contamination. The cDNA synthesis was carried out using 500 ng of RNA in accordance with the TaKaRa Synthesis protocol (Kusatsu, Japan). All qRT-PCR reactions were performed in triplicate to ensure the reproducibility of the data. In addition, the stability of the reference gene selected for normalization was confirmed across all samples by preliminary analysis, further supporting the reliability of the quantitative results.

### 2.3. qRT-PCR

The expression of *HCCAT5* was investigated with the qRT-PCR technique, through the utilization of the LightCycler Real-Time PCR (Roche Molecular Systems, Pleasanton, United States) and the standard SYBR Green Master Mix (Amplicon, Odense, Denmark). To conduct the investigation, specific forward and reverse primers for *HCCAT5* and *β*-actin were employed (Table 1).

### 2.4. Statistical Analysis

The relative expression of *HCCAT5* in gastric tumors and adjacent nontumor tissue samples was determined using the comparative Ct method (2-*Δ*Ct method) with the aid of Microsoft Excel software in 2021. SPSS Statistics Version 24 and GraphPad Prism 7 software (SPSS Inc, Chicago, United States) were employed to assess the correlation between *HCCAT5* expression and clinicopathological characteristics, utilizing Mann–Whitney and one-way ANOVA tests. The prognostic biomarker capability of *HCCAT5* was evaluated using ROC curve analysis. Statistically significant disparities were identified when the *p* value was less than 0.05.

## 3. Results

### 3.1. Samples

Among the total of 100 patients, 53 (53.0%) were identified as male, while the remaining 47 (47.0%) were identified as female. The mean age of the participants included in this research was 55 years. Among the patients, 46 individuals (46.0%) had a tumor size that measured less than 5 cm, whereas 54 patients (54.0%) had a tumor size exceeding 5 cm. Around 59.0% of the patients in the study exhibited the intestinal subtype, while the rest were diagnosed with the diffuse subtype. There were 52 patients (52.0%) classified as Stage I or II, while 48 patients (48.0%) were categorized as Stage III or IV. In 41% of patients, the presence of *Helicobacter pylori* (*H. pylori*) infection was detected, while 59% of patients tested negative for the infection. The clinicopathological characteristics of the patient have been examined in Table 2.

### 3.2. *HCCAT5* Expression in Gastric Tumors

**Figure 1:**
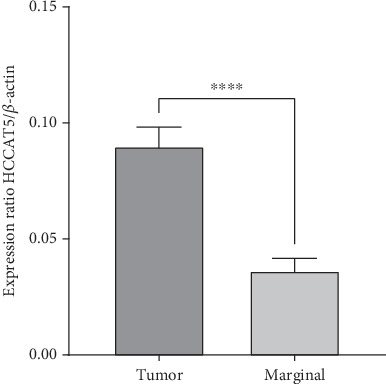
The expression of lncRNA *HCCAT5* in GC tumors is compared to that in adjacent nontumor tissues.

*HCCAT5* exhibited a notable increase in expression levels in cancerous tissues in comparison to adjacent nontumor tissue samples (*p* < 0.0001) (Figure 1).

### 3.3. Relationship Between *HCCAT5* Expression and Clinicopathological Characteristics

Table 2 illustrates a concise overview of the relationship between clinicopathological features and *HCCAT5* levels in individuals diagnosed with GC. There was no statistically significant correlation observed between the expression of *HCCAT5* and tumor size (*p* = 0.822), gender (*p* = 0.890), age (*p* = 0.340), TNM stage (*p* = 0.562), Lauren subtype (*p* = 0.568), lymph node metastasis (*p* = 0.148), and *H. pylori* infection (*p* = 0.758).

### 3.4. The ROC Analysis

**Figure 2:**
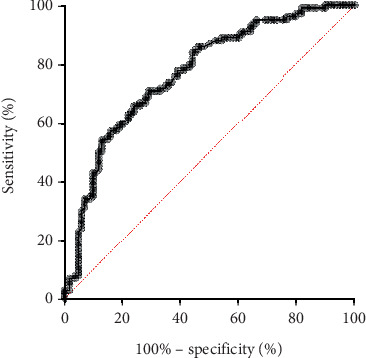
The specificity and sensitivity values obtained from the ROC curve analysis were 72% and 69%, respectively, with an AUC of 0.7686.

The sensitivity and specificity percentages derived from the ROC curve analysis were 69% and 72%, correspondingly. The ROC curve's area was 0.7686, as depicted in Figure 2 and Table 3.

## 4. Discussion

GC is a highly lethal form of solid tumor, contributing significantly to global rates of morbidity and mortality [29]. Despite showing a slight decrease in morbidity and mortality over the course of several decades [30], GC continues to pose a significant clinical obstacle due to its limited detection methods and unfavorable prognosis [31]. The identification of distinct biomarkers for the early detection, monitoring of therapeutic progress, and prognostic assessment of the condition could potentially enhance the chances of survival.

Hence, it is crucial to understand the processes and routes that result in the development and advancement of GC in order to pinpoint biomarkers that are efficient in early detection and therapy [32]. In recent times, several investigations have brought to light the involvement of lncRNAs in diverse cellular and molecular mechanisms. These lncRNAs exhibit a specific expression pattern in different cell types and are found to be localized within specific subcellular compartments. They play a crucial role in regulating alternative splicing, modifying chromatin, and controlling various stages of gene transcription, including mRNA activity, stability, and degradation mechanisms. Additionally, it has been suggested that the dysregulation of their expression takes place during the developmental phases of cancer [33, 34].

The present study first demonstrated that there is a high level of *HCCAT5* expression in tumor tissues in comparison to peripheral noncancerous tissues of patients with GC, suggesting a potential oncogenic role for *HCCAT5* in these individuals. In examining the relationship between elevated levels of *HCCAT5* expression and clinical pathological characteristics like age, gender, Lauren classification, tumor size, lymph node metastasis, TNM stage, and *H. pylori*, no statistically significant association was identified so that this phenomenon could be attributed to the specific demographic under investigation and the restricted sample size of the patients examined. *HCCAT5* may function as a biomarker in people with GC. Analysis of gene expression has revealed that *HCCAT5* exhibits specific expression patterns in particular tumor types, with a notably elevated expression level observed in hepatocellular carcinoma. Suppression of endogenous *HCCAT5* expression in the HepG2 malignant liver cell line through the use of small interfering RNA has been documented to reduce cell proliferation. The capacity to induce tumor formation in nude mice and alter the expression levels of certain genes associated with apoptosis indicates the potential significance of *HCCAT5* in the advancement and progression of hepatocellular carcinoma, with its mechanism potentially linked to apoptosis [27, 28]. Liu et al. demonstrated that the expression of the *HCCAT5* gene is detected in over 50% of liver malignancies. Additionally, this gene exhibits moderate expression in a limited number of tumor types, including colon cancer, GC, glioma, and lung cancer. However, it is important to note that no expression of the *HCCAT5* gene was observed in any normal tissue [28]. The expression pattern of *HCCAT5* suggests that it could serve as a promising molecular target for the advancement of novel cancer therapies while minimizing the potential for adverse reactions. *HCCAT5* stands out even more in the diagnosis of hepatocellular carcinoma due to its unique low expression rate in other cancer types. Inhibition of endogenous *HCCAT5* expression in malignant HepG2 hepatocytes using small interfering RNA results in the suppression of hepatocellular carcinoma cell proliferation. This suggests that the upregulation of *HCCAT5* gene transcription is crucial for the viability of hepatocellular carcinoma cells and is a consistent occurrence during tumorigenesis [28].

lncRNAs continue to serve as a remarkably stable reservoir of disease biomarkers within the human body [35]. The advent of genome-wide sequencing techniques has become a significant technological advancement, leading to the discovery of a vast number of dysregulated lncRNAs. These findings hold great promise for the potential utilization of lncRNAs in the diagnosis and prognosis of GC [36]. The presence of deregulated lncRNAs, including *H19*, *MALAT1*, *HOTAIR*, *GAPLINC*, *ANRIL*, *HULC*, *BANCR*, and *FENDRR*, has been observed in samples of GC [37]. Plasma analysis has revealed the presence of certain lncRNAs such as *UCA1*, *H19*, and *LINC00152*, which hold promise as noninvasive diagnostic indicators for individuals suffering from GC [38, 39]. Furthermore, aside from elucidating the function of lncRNAs in diagnosis, certain lncRNAs like *H19*, *UCA1*, *HOTAIR*, *TINCR*, and *PVT1* have the potential to serve as prognostic indicators [37]. lncRNAs play a crucial role in tumorigenicity by functioning as chromatin modulators, transcription regulators, regulators of mRNA stability or translation, and miRNA sponges [40]. Among these functions, the modulation of chromatin modification is particularly significant, and several lncRNAs have been identified as key players in this process [40]. These lncRNAs interact with histone modifiers, including TUG1, LINC00668, HOXA11-AS, LINC00152, GClnc1, and PVT1, to regulate chromatin structure and gene expression [37]. Additionally, lncRNAs can exert control over the expression of various epigenetic-modifying enzymes, such as DNMTs and PRC2, which are known to be involved in the development of cancer. Notably, certain lncRNAs, such as *ecCEBPA*, *MLK7-AS1*, *SNHG1*, and *AK058003*, interact with *DNMT* and have been implicated in the progression of GC [41–44]. The results underscore the varied and complex functions of lncRNAs in the realm of cancer biology, offering valuable perspectives on potential targets for cancer therapy. Consequently, pinpointing crucial molecules implicated in GC's progression can greatly enhance the timely diagnosis and control of the illness. Likewise, our research indicates that *HCCAT5* has the potential to serve as a valuable diagnostic biomarker for individuals with GC.

## 5. Conclusion

*HCCAT5* exhibits a distinct expression profile in cancer, particularly in GC. By promoting the proliferation of GC cells, *HCCAT5* plays a crucial role in the onset and advancement of tumorigenesis, suggesting its utility as a biomarker for early detection of GC.

## Figures and Tables

**Table 1:** The primers employed in this investigation.

**Gene**	**Forward**	**Reverse**
*HCCAT5*	5⁣′-TCTCCTGCAAACAGACAGCA-3⁣′	5⁣′-GCCAGGAGGGCATTATGGT-3⁣′
*β-Actin*	5⁣′-AGAGCTACGAGCTGCCTGAC-3⁣′	5⁣′-AGCACTGTGTTGGCGTACAG-3⁣′

**Table 2:** Association between the expression levels of *HCCAT5* and clinicopathological characteristics.

**Characteristics**	**No. of patients**	**p** ** value**
Age (years)		
≤ 55	58	0.340
> 55	42	
Gender		
Male	53	0.890
Female	47	
Lauren classification		
Intestinal	59	0.568
Diffuse	41	
Tumor size (cm)		
< 5 cm	46	0.822
≥ 5 cm	54	
Lymph node metastasis		
Positive	40	0.148
Negative	60	
TNM stage		
I, II	52	0.562
III, IV	48	
*Helicobacter pylori*		
Positive	41	0.758
Negative	59	

**Table 3:** The diagnostic performance of lncRNA *HCCAT5* in gastric cancer was assessed through statistical analysis of its ROC curve.

**The ROC curve data**	**Values**
AUC	0.7686
Sensitivity (%)	69
Specificity (%)	72
Cutoff score	> 0.0055
Std. error	0.6749–0.7489
95% confidence interval	< 0.0017

## Data Availability

The data that support the findings of this study are available from the corresponding author upon reasonable request.
